# Predictors of weight reduction effectiveness of SGLT2 inhibitors in diabetes mellitus type 2 patients

**DOI:** 10.3389/fendo.2023.1251798

**Published:** 2024-01-24

**Authors:** Pojsakorn Danpanichkul, Worapaka Manosroi, Tharadon Nilsirisuk, Theetouch Tosukhowong

**Affiliations:** ^1^ Immunology Unit, Department of Microbiology, Faculty of Medicine, Chiang Mai University, Chiang Mai, Thailand; ^2^ Division of Endocrinology, Department of Internal Medicine, Chiang Mai University, Chiang Mai, Thailand; ^3^ Clinical Epidemiology and Clinical Statistics Center, Faculty of Medicine, Chiang Mai University, Chiang Mai, Thailand; ^4^ Faculty of Medicine, Chiang Mai University, Chiang Mai, Thailand; ^5^ Department of Physiology, Faculty of Medicine, Chiang Mai University, Chiang Mai, Thailand

**Keywords:** SGLT2 inhibitors, weight reduction, diabetes mellitus type 2, weight loss, diabetes

## Abstract

**Background:**

Sodium-glucose cotransporter-2 inhibitors (SGLT2i) are a novel medication for treating type 2 diabetes (T2DM), which have the pleiotropic effect of weight reduction. This study aimed to evaluate clinical and biochemical predictors of effective weight reduction in T2DM patients who use various types of SGLT2i.

**Materials and methods:**

A retrospective study was conducted with 289 adults diagnosed with T2DM who were first prescribed SGLT2i either as monotherapy or add-on therapy. The primary outcome was the identification of clinical and biochemical factors that can potentially induce meaningful weight reduction (>3% in 1 year) in T2DM patients while using SGLT2 inhibitors. The relationship between predictors and significant weight loss was assessed using logistic regression analysis, including adjustment for confounding factors. Results are presented as odds ratios (ORs) with a 95% confidence interval (CI).

**Results:**

Among the 289 patients, 45.6% had significant weight loss following SGLT2i use. The significant clinical predictors were age >70 years old (OR 3.26, 95% CI 1.39-7.6, p=0.006), body mass index >25 kg/m^2^ (OR 1.02, 95% CI 1.01-1.05, p=0.049), and the use of sulfonylureas (OR 2.41, 95% CI 1.15-5.09, p=0.020). Additionally, the use of HCTZ showed significantly decreased odds of weight loss (OR 0.35, 95% CI 0.13-0.96, p=0.043).

**Conclusion:**

This research highlights multiple clinical factors that potentially can predict meaningful weight loss in patients with T2DM who are treated with SGLT2i. These findings could facilitate the identification of patients who might benefit from the weight loss effects of SGLT2i.

## Introduction

The escalating prevalence of metabolic syndrome is a cause of concern as it indicates a heightened risk of numerous health conditions ranging from cardiovascular complications to malignancy that are associated with an elevated mortality rate. The two most essential components of metabolic syndrome are diabetes mellitus type 2 (T2DM) and obesity, which is now defined by the new term “diabesity” (1–4). Recently, various novel antidiabetic drugs e.g. dual GIP/GLP-1 receptor co-agonist, GLP-1 receptor agonist and sodium-glucose cotransporter-2 inhibitors (SGLT2i) provided benefits in weight reduction (5–7). Sodium-glucose cotransporter-2 inhibitors (SGLT2i) have emerged as a significant advance in the management of T2DM (7). This novel class of antidiabetic drugs exhibits unique insulin-independent action by inhibiting renal glucose reabsorption in the proximal renal tubules, which leads to lower blood glucose levels (8). Apart from its glucose-lowering effect, SGLT2i has shown pleiotropic effects, including cardiovascular and renal benefits, blood pressure reduction, and body weight reduction. Also, this class of medication has been approved in various countries for use in non-diabetes patients with heart failure or chronic kidney disease. Reported side effects of SGLT2i include urinary tract infections, genital tract infection and euglycemia diabetes ketoacidosis (9).

Given the benefit of SGLT2i on the reduction of body weight, it may be helpful in T2DM patients who are overweight or obese, especially patients taking other medications that can cause weight gain. SGLT2i has been shown to result in a slight reduction in body weight compared to placebo. A meta-analysis of 43 randomized controlled trials comparing SGLT2i with placebo has reported that a mean reduction of 1.88 kg of body weight was observed in SGLT2i users compared to placebo (10). The potential mechanisms of weight reduction from SGLT2i are believed to result from an increase in the urinary glucose excretion (11). However, initially, fluid loss may play a role. Overall reductions in body weight are believed to be due to caloric loss and the activation of lipolysis, which results in fat loss (12, 13).

Patients taking SGLT2i have shown highly variable weight loss ranging from 2-4% of initial body weight (14). Moreover, the weight-lowering effect is unpredictable and could result from intricate interactions between non-biological and biological factors (15). A previous study demonstrated that multiple factors can predict meaningful weight reduction in T2DM patients using SGLT2i, including regular exercise, normal renal function, and concurrent use of metformin. That study, however, included only one type of SGLT2i, dapagliflozin (16).

A more precise understanding of the predictors of weight reduction in SGLT2i users would optimize its use, maximizing benefits, minimizing potential adverse effects, and providing an efficient treatment strategy for using SGLT2i among patients with T2DM. The present study aimed to demonstrate clinical and biochemical predictors of effective weight reduction in T2DM patients who use various types of SGLT2i.

## Materials and methods

This retrospective cohort study included adults aged 18 years and older with T2DM who were first prescribed SGLT2i either as monotherapy or add-on therapy at the Internal Medicine Department, Faculty of Medicine, Chiang Mai University, between 1 January 2018 and 31 December 2022. This study adhered to the principles of the Declaration of Helsinki. The Institutional Review Board of the Faculty of Medicine Chiang Mai University approved the study (MED 2566-09418), which waived the requirement for informed consent because of the retrospective nature of the study.

The study excluded patients who used GLP1-RA, was diagnosed with type 1 diabetes mellitus, had a recent history of herb use or use of any other drugs with a steroid component, had a history of steroid treatment, had a duration of follow-up <1 year, were pregnant, had chronic kidney disease (CKD) requiring peritoneal dialysis or hemodialysis, had undergone bariatric surgery or used of other weight-reduction agents, and cases where complete physical examination and laboratory investigation data could not be extracted from the medical records. Follow-up time was defined as the interval from the starting date of SGLT2i treatment to the points of interest at 6 and 12 months. Significant weight reduction was defined as >3% weight loss in 12 months. Height was measured to the nearest 0.1 cm using a stadiometer. Body weight was measured to the nearest 0.1 kg using a balance scale, with the participants wearing light clothing.

Data obtained from medical records included demographic information, e.g., age and gender, medical history including comorbidities (dyslipidemia, hypertension, atherosclerotic cardiovascular disease (ASCVD), CKD, nonalcoholic fatty liver disease (NAFLD), and other comorbidities). Histories of alcohol consumption and tobacco use were recorded, as well as current medication usage, including types of diabetes drugs, lipid-lowering agents, and anti-hypertensive agents. Anthropometric measurements, including weight, height, and body mass index (BMI), were collected along with systolic and diastolic blood pressure readings. Biochemical parameters were documented, including HbA1c and fasting blood glucose levels, total cholesterol, low-density lipoprotein (LDL) and high-density lipoprotein (HDL) levels, and creatinine levels with estimated glomerular filtration rates (eGFR). Anthropometric measurements and biochemical parameters were determined at baseline and 6 and 12 months.

### Statistical analysis

The data were analyzed using STATA program version 17.0. Statistical significance was defined as two-tailed with a p-value <0.05. For categorical variables, counts and percentages are reported; for normally distributed continuous variables, means and standard deviations (SD) are presented. For non-normally distributed continuous variables, medians with interquartile ranges (IQR) are shown. For continuous data, univariable analysis was used with the independent t-test for normally distributed variables, and the Wilcoxon rank-sum test was employed for non-normally distributed variables. Additionally, multivariable logistic regression analyses of the predictive factors for >3% weight loss at one year were performed and are reported as odds ratios (OR) with a 95% confidence interval (CI). To calculate the sample size for the multivariate analysis, an empirical rule was used based on the estimation of 10 effects for each independent variable considered for inclusion in the model. As the study planned to include at least ten predictors, it was assumed that 40% of patients would achieve a >3% weight loss one year after SGLT2i use and a 25% withdrawal from the study; at least 250 patients needed to be included to identify the predictors.

## Results

A total of 289 T2DM patients were included in the study and were categorized into those who experienced significant weight loss (>3% in 1 year) and those who had no significant weight loss. The majority of the cohort were males (n=166, 57.6%). The median age was 65 years (IQR 59-71). The median BMI was 25.8 kg/m^2^ (IQR 23.4-29). The median HbA1c was 7.5% (IQR 6.8-8.5). The median weight reduction at 1 year was -2 (IQR -4,0) kg. Of the participants, 45.6% (n=132) achieved a significant weight loss, while 54.4% (n=157) had no significant weight loss. Median weight reduction at one year in the significant weight loss group was -4 kg (IQR -6,-3), while in the non-significant weight loss group, the weight reduction was 0 kg (IQR -1, 1.2). The difference in weight loss between the two groups was statistically significant. Except for body weight change at 12 months, there were no statistically significant differences between the two groups in other demographic data, including sex, age, BMI, blood pressure, baseline body weight, underlying diseases, and the use of antidiabetic agents and antihypertensive medications. Additionally, there was no significant difference between the groups in the type and dosage of SGLT2i. The most used type of SGLT2i was empagliflozin (n=153, 53%), followed by dapagliflozin (n=94, 32.5%). Baseline biochemical investigations, including HbA1c levels, creatinine and total cholesterol, LDL, and HDL, were similar between the groups. Changes in HbA1c at 12 months and the number of patients with creatinine rising >30% after SGLT2i were also comparable (**Table 1**).

**Table 1:** Baseline characteristics (n=289).

Demographic data	Significant weight loss (>3% in 1 year) (n=132)	No significant weight loss (n=157)	P-value
Male, n (%)	79 (59.8)	87 (55.8)	0.487
Age (IQR, years)	66.5 (59-72.5)	65 (60-71)	0.556
BMI (IQR, kg/m^2^)	26.2 (23.8-29.4)	25.7 (23.3-28.8)	0.751
Baseline body weight (IQR, kg)	69 (60.7-79)	66.7 (59-77)	0.134
Body weight at 6 months (IQR, kg)	64 (57-74)	67 (60-78)	0.193
Body weight at 12 months (IQR, kg)	66.8 (58.5-74.8)	68 (44.7-79.7)	0.242
Body weight changes at 12 months (IQR, kg)	-4 (-6,-3)	0 (-1, 1.2)	<0.001
Systolic blood pressure (IQR, mmHg)	135 (122-144)	133 (124-142)	0.906
Diastolic blood pressure (IQR, mmHg)	74 (67-83)	75 (68-81)	0.416
Underlying disease, n (%)			
- Hypertension	132 (84.1)	110 (83.3)	0.865
- Dyslipidemia	131 (83.4)	116 (87.8)	0.286
- ASCVD	27 (20.4)	26 (16.6)	0.394
- NAFLD	4 (3.0)	7 (4.5)	0.527
- CKD	21 (15.9)	24 (15.3)	0.884
Smoker, n (%)	7 (5.3)	6 (3.8)	0.545
Anti-diabetes medication, n (%)			
- Metformin	106 (80.3)	118 (75.2)	0.297
- Sulfonylureas	52 (39.4)	59 (37.6)	0.752
- Pioglitazone	23 (17.4)	37 (23.6)	0.200
- DPP-4 inhibitor	74 (56.1)	87 (55.4)	0.912
- Insulin	18 (13.6)	26 (16.6)	0.491
SGLT2i, n (%)			
- Empagliflozin 25 mg	35 (26.5)	45 (28.7)	
- Empagliflozin 10 mg	34 (25.7)	39 (24.8)	
- Dapagliflozin 10 mg	39 (29.5)	50 (31.8)	
- Dapagliflozin 5 mg	3 (2.3)	2 (1.3)	
- Luseogliflozin 5 mg	11 (8.4)	10 (6.4)	
- Luseogliflozin 2.5 mg	6 (4.5)	11 (7)	
- Canagliflozin 300 mg	1 (0.8)	0 (0)	
- Canagliflozin 100 mg	3 (2.3)	0 (0)	0.224
Antihypertensive medication, n (%)			
- ACEI or ARBs	72 (54.6)	94 (59.9)	0.362
- Calcium channel blockers	50 (37.9)	72 (45.9)	0.171
- Hydrochlorothiazide	14 (10.6)	24 (15.3)	0.241
- Beta-blockers	37 (28.1)	52 (33.1)	0.350
- Mineralocorticoid receptor blockers	7 (5.3)	12 (7.6)	0.424
Biochemical investigation			
HbA1c (IQR, %)	7.4 (6.9-8.4)	7.5 (6.7-8.7)	0.805
HbA1c at 6 months (IQR, %)	6.9 (6.4-7.7)	6.9 (6.4-7.6)	0.269
HbA1c at 12 months (IQR, %)	7 (6.6-7.9)	7.1 (6.6-7.6)	0.664
HbA1c changes at 12 months (IQR, %)	-0.3 (-1.3, 0.28)	-0.2 (-1.2,0.44)	0.430
Creatinine (IQR, mg/dL)	0.9 (0.7-1.26)	0.9 (0.7-1.3)	0.389
Total cholesterol (IQR, mg/dL)	164 (136-188)	148 (125-190)	0.422
LDL (IQR, mg/dL)	93 (73-113)	85 (68-127)	0.758
HDL (IQR, mg/dL)	49 (41-59)	47 (39-57)	0.701
Patients with creatinine rising >30% after SGLT2i, n (%)	60 (45.5)	72 (45.9)	0.945

IQR, Interquartile range; BMI, Body mass index; ASCVD, Atherosclerotic cardiovascular disease; CKD, Chronic kidney disease; NAFLD, Non-alcoholic fatty liver disease; DPP-4 inhibitor, Dipeptidyl peptidase-4 inhibitor; ACEI, Angiotensin converting enzyme inhibitors; ARBs, Angiotensin receptor blockers; LDL, Low-density lipoprotein; HDL, High-density lipoprotein.

In a multivariable analysis exploring the potential predictors of significant weight loss after SGLT2i use, age over 70 years was found to be associated with a significantly higher likelihood of weight loss (OR 3.26, 95% CI 1.39-7.6, p=0.006). Baseline BMI >25 kg/m^2^ also showed a small but statistically significant association with weight loss (OR 1.02, 95% CI 1.01-1.05, p=0.049). The use of sulfonylureas was statistically significantly associated with increased weight loss (OR 2.41, 95% CI 1.15-5.09, p=0.020), while hydrochlorothiazide (HCTZ) use was associated with decreased odds of weight loss (OR 0.35, 95% CI 0.13-0.96, p=0.043). No statistically significant associations with weight loss were found for sex, baseline blood pressure, NAFLD and ASCVD status, smoking status, the maximum dose of SGLT2i use, the use of other medications (e.g., metformin, pioglitazone, DPP-4 inhibitors, insulin, and calcium channel blockers), baseline HbA1c, changes in HbA1c, eGFR, or patients with an increase in creatinine of >30% after SGLT2i (**Table 2**).

**Table 2:** Multivariable analysis of predictors for significant weight loss after using SGLT2i.

Predictor	OR (95% CI)	p-value
Age >70 years	3.26 (1.39-7.6)	0.006
Male	0.81 (0.35-1.90)	0.642
Baseline BMI >25 kg/m^2^	1.02 (1.01-1.05)	0.049
Baseline systolic blood pressure	0.99 (0.96-1.01)	0.429
Baseline diastolic blood pressure	0.98 (0.95-1.01)	0.632
NAFLD	0.55 (0.11-2.63)	0.463
ASCVD	1.22 (0.50-2.95)	0.650
Smoker	2.96 (0.55-15.80)	0.203
Patients who used maximum dose of SGLT2i	1.22 (0.61-2.44)	0.571
Metformin use	1.41 (0.53-3.71)	0.480
Sulfonylureas use	2.41 (1.15-5.09)	0.020
Pioglitazone use	0.66 (0.26-1.67)	0.391
DPP-4 inhibitor use	0.65 (0.31-1.36)	0.262
Insulin use	0.99 (0.40-2.42)	0.988
HCTZ use	0.35 (0.13-0.96)	0.043
Calcium channel blocker use	0.78 (0.40-1.54)	0.479
Baseline HbA1c	1.03 (0.74-1.42)	0.855
HbA1c changes	0.94 (0.71-1.25)	0.700
eGFR	0.44 (0.16-1.22)	0.116
Patients with creatinine rising >30% after SGLT2i	1.08 (0.51-2.29)	0.827

BMI, Body mass index; ASCVD, Atherosclerotic cardiovascular disease; NAFLD, Non-alcoholic fatty liver disease; DPP-4 inhibitor, Dipeptidyl peptidase-4 inhibitor; HCTZ, Hydrochlorothiazide; eGFR, Estimated glomerular filtration rate.

## Discussion

This retrospective cohort study highlights an interesting finding: there are multiple clinical and biochemical indicators that could potentially assist in predicting significant weight loss in patients with T2DM following treatment with SGLT2i. These factors are age over 70 years, baseline BMI >25 kg/m^2^, sulfonylureas users, and HCTZ non-users. These findings suggested the existence of certain patterns that could facilitate the selection of patients who might respond to the weight loss benefits of SGLT2i and could help optimize the use of SGLT2i for weight management in T2DM patients.

In congruence with previous studies, approximately half (45.6%) the T2DM patients in the present study versus 61% in a previous study had significant weight loss after using SGLT2i (16). The previous study reported that regular exercise, normal renal function and the use of metformin were predictors of weight loss in SGLT2i users (16). Unlike in this present study, having normal renal function and using metformin had no significant association with weight reduction. The previous study also reported no significant association between significant weight reduction and older age, baseline BMI >25 kg/m^2^, sulfonylureas use, or non-use of HCTZ. However, only one type of SGLT2i (dapagliflozin) was included in the previous study, while 4 types of SGLT2i were included in the present study. In addition, the previous study did not report whether pioglitazone or glucagon-like peptide 1 (GLP-1) were included in the cohort, two glucose-lowering agents that may affect weight change. Additionally, changes in HbA1c level and increases of serum creatinine which can indicate fluid loss were not considered in that study. A further larger and more thorough cohort study is needed to explore these aspects more fully.

Intriguingly, there was a significant association of age over 70 years with weight loss following SGLT2i. This suggests that older patients might benefit more in terms of weight loss when treated with SGLT2i, an association which could possibly be explained by higher susceptibility to the calorie-reducing effects of SGLT2i. Physiologically, adults aged less than 70 years old had a loss of fat-free mass, especially skeletal muscle, and an increase in fat mass (17, 18). However, after the age of 70-80 years, a trend to a reduction of fat mass was observed (18). As mentioned earlier, the late phase of weight loss from SGLT2i can be explained by fat loss, so the physiologic changes in the elderly over 70 years could explain the association with significant weight reduction in individuals who used SGLT2i. Another factor that could contribute to this finding was that elderly patients tended to have higher adherence to diabetes medications than younger patients, resulting in more clinical benefits, e.g., the greater weight reduction with SGLT2i observed in the elderly than in younger patients (19). However, this cannot be confirmed by the present study as data regarding medication adherence was not reported in this cohort. Larger scale studies are required to understand the exact mechanisms behind this association.

Patients with a baseline BMI >25 kg/m^2^ showed a marginally statistically significant association with weight loss, with an OR of 1.02. Based on WHO criteria for Asian individuals, a BMI over 25 kg/m^2^ was defined as obesity (20). The physiologic effect of SGLT2i per se may not fully explain this association. A potential underpinning theory could be that T2DM patients with obesity may have more enthusiasm for lifestyle modification (e.g., performing strenuous exercise, strict diet control with caloric restriction) than those with a normal BMI. Data regarding lifestyle modification were not obtained for this cohort. The p-value of this factor showed a marginally significant association with meaningful weight loss. However, the OR of this factor was very small, and the lower CI almost crossed 1, so this association may not have any clinical significance.

Interestingly, the use of sulfonylureas, a widely used medication in diabetes management, together with SGLT2i, was found to be associated with a higher degree of weight loss. Sulfonylureas stimulate the release of insulin from the pancreas. One of the most common side effects of sulfonylureas is a weight gain of approximately 2.0-2.3 kg (21). Mechanisms of weight gain from sulfonylureas include 1) hypoglycemia, which can induce increased caloric intake, and 2) increased insulin level, which can lead to lipogenesis and cause excess fat deposition (21). Thus, it could be presumed that this excess fat deposition could be reduced by the effects of SGLT2i, which can promote fat loss in the latter phase of the weight reduction mechanism.

The use of HCTZ, a diuretic often used to manage hypertension which has weight loss as a side effect, has been linked with decreased odds of weight loss (22, 23). However, the explanation of the relationship between the comedication of HCTZ and SGLT2i needs to be clarified as duration, dosage, and patient underlying comorbidities need to be considered. One plausible mechanism is that because HCTZ has been reported to be associated with insulin resistance and an increase in visceral adipose tissue, this population tended to have a high incidence of metabolic syndrome and obesity, which may have resulted in resistance to SGLT2i-induced weight loss (24).

Strengths of this study include that, unlike a previous study (16), it investigated various types of SGLT2i, making the results of the present study more generalizable. This study also used multivariable logistic regression analysis incorporating multiple confounders for adjustment, which made the results more interpretable and more accurate. The results highlight the potential role of individual characteristics and concurrent medication used in determining weight loss outcomes with SGLT2i therapy. These predictors could help clinicians identify which patients will benefit from the extra-glycemic effect of SGLT2i, assist in maximizing the usage of SGLT2i, and offer patients with T2DM an effective SGLT2i treatment plan.

Several limitations need to be mentioned, including the retrospective study design and the reliance on patient records, which may have yet to capture all relevant data, e.g., data on lifestyle activities, diet control, calorie intake and calories used per day which could affect the outcome (25). In addition, the findings may not be applicable to all patient populations due to the specific inclusion criteria of the study and the fact that the study population was primarily comprised of Asians, who may exhibit differential effects in response to SGLT2i and T2DM pathophysiology (26, 27). Body composition was not measured in this cohort, so the underpinning mechanism of weight reduction could not be fully elucidated. Further prospective studies conducted in different ethnicities, measuring body composition, and including data that could interfere with outcomes are needed to confirm these findings and further elucidate the underlying mechanisms.

## Conclusion

This study identified multiple potential predictors of significant weight loss following SGLT2i therapy in T2DM patients, including advanced age, higher BMI, sulfonylureas use, and HCTZ non-use. These findings could help optimize the use of SGLT2i and provide an efficient treatment strategy for using SGLT2i for patients with T2DM. Further studies are needed to confirm these findings and elucidate the underlying mechanisms.

## Data availability statement

The raw data supporting the conclusions of this article will be made available by the authors, without undue reservation.

## Ethics statement

The studies involving humans were approved by Faculty of Medicine, Chiang Mai University. The studies were conducted in accordance with the local legislation and institutional requirements. The ethics committee/institutional review board waived the requirement of written informed consent for participation from the participants or the participants’ legal guardians/next of kin because the retrospective nature of the study.

## Author contributions

WM designed the study, supervised the study, analyzed and interpreted the data and was the major contributor in writing and editing the manuscript. PD performed the data curation, data validation, analyzed and wrote the original draft of manuscript. TT and TN performed the data curation of the study. All authors contributed to the article and approved the submitted version.

## References

[1] Swinburn BA Sacks G Hall KD McPherson K Finegood DT Moodie ML. The global obesity pandemic: shaped by global drivers and local environments. Lancet. (2011) 378(9793):804–14. doi: 10.1016/S0140-6736(11)60813-1 21872749

[2] Abudawood M. Diabetes and cancer: A comprehensive review. J Res Med Sci (2019) 24:94. doi: 10.4103/jrms.JRMS_242_19 31741666 PMC6856544

[3] Upadhyay J Farr O Perakakis N Ghaly W Mantzoros C. Obesity as a disease. Med Clin North Am (2018) 102(1):13–33. doi: 10.1016/j.mcna.2017.08.004 29156181

[4] Leitner DR Frühbeck G Yumuk V Schindler K Micic D Woodward E. Obesity and type 2 diabetes: two diseases with a need for combined treatment strategies - EASO can lead the way. Obes Facts. (2017) 10(5):483–92. doi: 10.1159/000480525 PMC5741209 29020674

[5] Chavda VP Ajabiya J Teli D Bojarska J Apostolopoulos V. Tirzepatide, a new era of dual-targeted treatment for diabetes and obesity: A mini-review. Molecules. (2022) 27(13):1–10. doi: 10.3390/molecules27134315 PMC9268041 35807558

[6] Trujillo JM Nuffer W Smith BA. GLP-1 receptor agonists: an updated review of head-to-head clinical studies. Ther Adv Endocrinol Metab (2021) 12:2042018821997320. doi: 10.1177/2042018821997320 33767808 PMC7953228

[7] Saisho Y. SGLT2 inhibitors: the star in the treatment of type 2 diabetes? Diseases (2020) 8(2):1–12. doi: 10.3390/diseases8020014 32403420 PMC7349723

[8] Vallon V. The mechanisms and therapeutic potential of SGLT2 inhibitors in diabetes mellitus. Annu Rev Med (2015) 66:255–70. doi: 10.1146/annurev-med-051013-110046 25341005

[9] Patel DK Strong J. The pleiotropic effects of sodium-glucose cotransporter-2 inhibitors: beyond the glycemic benefit. Diabetes Ther (2019) 10(5):1771–92. doi: 10.1007/s13300-019-00686-z PMC6778563 31456166

[10] Pereira MJ Eriksson JW. Emerging role of SGLT-2 inhibitors for the treatment of obesity. Drugs. (2019) 79(3):219–30. doi: 10.1007/s40265-019-1057-0 PMC6394798 30701480

[11] DeFronzo RA Norton L Abdul-Ghani M. Renal, metabolic and cardiovascular considerations of SGLT2 inhibition. Nat Rev Nephrol. (2017) 13(1):11–26. doi: 10.1038/nrneph.2016.170 27941935

[12] Bolinder J Ljunggren ÖChecktae Johansson L Wilding J Langkilde AM Sjöström CD. Dapagliflozin maintains glycaemic control while reducing weight and body fat mass over 2 years in patients with type 2 diabetes mellitus inadequately controlled on metformin. Diabetes Obes Metab (2014) 16(2):159–69. doi: 10.1111/dom.12189 23906445

[13] Szekeres Z Toth K Szabados E. The effects of SGLT2 inhibitors on lipid metabolism. Metabolites. (2021) 11(2):2–9. doi: 10.3390/metabo11020087 PMC7912792 33535652

[14] Janež A Fioretto P. SGLT2 inhibitors and the clinical implications of associated weight loss in type 2 diabetes: A narrative review. Diabetes Ther (2021) 12(8):2249–61. doi: 10.1007/s13300-021-01104-z PMC8342745 34244976

[15] Brown E Wilding JPH Barber TM Alam U Cuthbertson DJ. Weight loss variability with SGLT2 inhibitors and GLP-1 receptor agonists in type 2 diabetes mellitus and obesity: Mechanistic possibilities. Obes Rev (2019) 20(6):816–28. doi: 10.1111/obr.12841 30972878

[16] Huh Y Kim YS. Predictors for successful weight reduction during treatment with Dapagliflozin among patients with type 2 diabetes mellitus in primary care. BMC Primary Care (2022) 23(1):134. doi: 10.1186/s12875-022-01748-1 35624416 PMC9137162

[17] Villareal DT Apovian CM Kushner RF Klein S. Obesity in older adults: technical review and position statement of the American Society for Nutrition and NAASO, The Obesity Society. Am J Clin Nutr (2005) 82(5):923–34. doi: 10.1093/ajcn/82.5.923 16280421

[18] Ding J Kritchevsky SB Newman AB Taaffe DR Nicklas BJ Visser M. Effects of birth cohort and age on body composition in a sample of community-based elderly. Am J Clin Nutr (2007) 85(2):405–10. doi: 10.1093/ajcn/85.2.405 17284736

[19] Afaya RA Bam V Azongo TB Afaya A Kusi-Amponsah A Ajusiyine JM. Medication adherence and self-care behaviours among patients with type 2 diabetes mellitus in Ghana. PloS One (2020) 15(8):e0237710. doi: 10.1371/journal.pone.0237710 32822381 PMC7446850

[20] Lim JU Lee JH Kim JS Hwang YI Kim TH Lim SY. Comparison of World Health Organization and Asia-Pacific body mass index classifications in COPD patients. Int J Chron Obstruct Pulmon Dis (2017) 12:2465–75. doi: 10.2147/COPD.S141295 PMC5571887 28860741

[21] Apovian CM Okemah J O'Neil PM. Body weight considerations in the management of type 2 diabetes. Adv Ther (2019) 36(1):44–58. doi: 10.1007/s12325-018-0824-8 30465123 PMC6318231

[22] Freis ED Reda DJ Materson BJ. Volume (weight) loss and blood pressure response following thiazide diuretics. Hypertension. (1988) 12(3):244–50. doi: 10.1161/01.HYP.12.3.244 3049338

[23] Douketis JD Sharma AM. The management of hypertension in the overweight and obese patient: is weight reduction sufficient? Drugs (2004) 64(8):795–803. doi: 10.2165/00003495-200464080-00001 15059036

[24] Eriksson JW Jansson PA Carlberg B Hägg A Kurland L Svensson MK. Hydrochlorothiazide, but not Candesartan, aggravates insulin resistance and causes visceral and hepatic fat accumulation: the mechanisms for the diabetes preventing effect of Candesartan (MEDICA) Study. Hypertension. (2008) 52(6):1030–7. doi: 10.1161/HYPERTENSIONAHA.108.119404 18981327

[25] Euser AM Zoccali C Jager KJ Dekker FW. Cohort studies: prospective versus retrospective. Nephron Clin Pract (2009) 113(3):c214–7. doi: 10.1159/000235241 19690438

[26] Lim LL Tan AT Moses K Rajadhyaksha V Chan SP. Place of sodium-glucose cotransporter-2 inhibitors in East Asian subjects with type 2 diabetes mellitus: Insights into the management of Asian phenotype. J Diabetes Complications. (2017) 31(2):494–503. doi: 10.1016/j.jdiacomp.2016.10.008 27866701

[27] Ke C Narayan KMV Chan JCN Jha P Shah BR. Pathophysiology, phenotypes and management of type 2 diabetes mellitus in Indian and Chinese populations. Nat Rev Endocrinol (2022) 18(7):413–32. doi: 10.1038/s41574-022-00669-4 PMC9067000 35508700

